# An Agouti-Signaling-Protein Mutation is Strongly Associated with Melanism in European Roe Deer (*Capreolus capreolus*)

**DOI:** 10.3390/genes11060647

**Published:** 2020-06-11

**Authors:** Monika Reissmann, Walburga Lutz, Dietmar Lieckfeldt, Edson Sandoval-Castellanos, Arne Ludwig

**Affiliations:** 1 Albrecht Daniel Thaer-Institute, Faculty of Life Sciences, Humboldt-University Berlin, 10099 Berlin, Germany; monika.reissmann@agrar.hu-berlin.de; 2 Wildlife Research Institute, 53229 Bonn, Germany; lutz@pscherrer.de; 3 Leibniz-Institute for Zoo & Wildlife Research, Department of Evolutionary Genetics, 10315 Berlin, Germany; lieckfeldt@izw-berlin.de; 4 Institute of Animal Breeding and Husbandry, Kiel University, 24098 Kiel, Germany; edsonsandovalc@outlook.com

**Keywords:** coat color, melanistic, cervid, ASIP, MC1R

## Abstract

Although the European roe deer (*Capreolus capreolus*) population of North-West Germany has a remarkable number of melanistic specimens between 10% and 25%, the underlying genetic mutation-causing melanism is still unknown. We used a gene targeting approach focusing on *MC1R* and *ASIP* as important genes of coat coloration. Overall, 1384 bp of *MC1R* and 2039 bp of *ASIP* were sequenced in 24 specimens and several SNPs were detected. But only the *ASIP*-SNP c.33G>T completely segregated both phenotypes leading to the amino acid substitution p.Leu11Phe. The SNP was further evaluated in additional 471 samples. Generally, all black specimens (*n* = 33) were homozygous TT, whereas chestnut individuals were either homozygote GG (*n* = 436) or heterozygote GT (*n* = 26). Considering the fact that all melanistic animals shared two mutated alleles of the strongly associated SNP, we concluded that melanism is inherited in a recessive mode in European roe deer.

## 1. Introduction

Melanism is a widespread phenomenon in animals. Dark phenotypes are identified in many groups of mammals including Felidae, Canidae, Cervidae and Bovidae but they are also present in insects, reptiles, birds, and other animals. Well-known melanistic animals are the black leopard (*Panthera pardus*) and the black jaguar (*Panthera onca*). In contrast to the black jaguar, where the black allele is dominant, the black mutation in leopards has a recessive mode of inheritance [1]. When compared to felids, melanistic specimens occur less frequently in cervids. In North American whitetail deer (*Odocoileus virginianus*) melanistic individuals are rarer than albinos ([2] and black-colored European red deer (*Cervus elaphus*) are also very rare. Particularly, the semi-domestic fallow deer (*Dama dama*) has a greater frequency of melanistic specimens which seems to be a product of artificial selection as often seen in domestic species [3]. The European roe deer (*Capreolus capreolus*) is the only cervid species which harbors a noteworthy natural population of melanistic specimens [4]. Although black European roe deer have been known in North-West Germany for at least several centuries and received great interest from royal houses in ancient times [5], they are less studied and even the knowledge about the genetic background of their melanism is still unknown.

**Figure 1:**
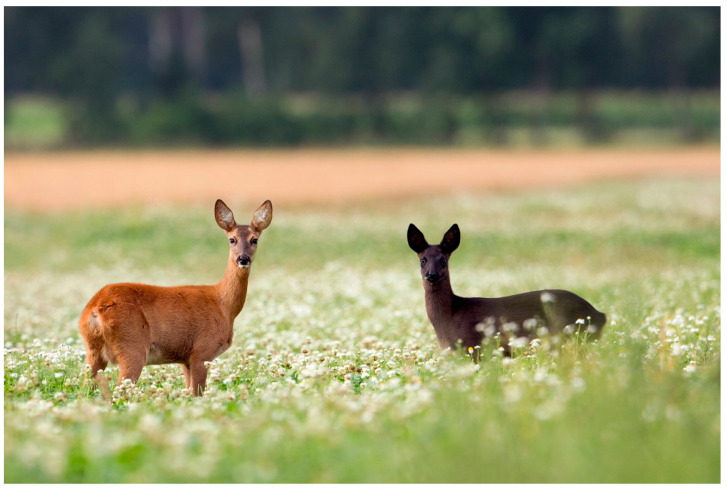
Chestnut and melanistic European roe deer (photo presented by Jan Piecha).

In mammals, basic coat coloration of hair and skin is defined by the ratio and distribution of the pigments eumelanin and pheomelanin [3]. Melanism is a mutation that increases the production of eumelanin in skin and hair. The darkish color of the eumelanin has a phenotypic expression from dark brown to black fur and skin [3]. In European roe deer, melanistic animals are characterized by a black coat and skin whereas the common phenotype ranges from reddish to chestnut (Figure 1).

Mutations in several genes are considered as causative mechanisms for melanism. In our study, we focused on the genes of the MC1R (melanocortin 1 receptor) and the ASIP (agouti-signaling protein) as target genes for melanism in European roe deer because of their prominent roles in pigment production [3,6,7,8].

## 2. Materials and Methods

### 2.1. Sampling and DNA Extraction

Tissue samples from 4 chestnut (Tierpark Berlin, Germany) and 20 black-colored individuals (North-West Germany and Saxony-Anhalt) were utilized for the identification of candidate mutations associated with melanism in European roe deer. Genomic DNA was extracted by incubating in 180 µL T1 buffer and 20 µL proteinase K (Macherey-Nagel, Berlin, Germany) followed by a salting out procedure [9].

### 2.2. Sequencing of Target Genes

The draft genome of European roe deer (>lcl|Deer_k43_scaffold143768:28399-31352 10.1, [10]) and the *MC1R* sequence of roe deer (Y13960) were utilized for the primer design of the *melanocortin 1 receptor (MC1R)* gene. For the *agouti-signaling protein* (*ASIP*) gene, no reference sequence was available from the cervid species. The draft version of the European roe deer genome (>lcl|Deer_k43_scaffold320422:2380-9700 13.5, [10]) was utilized for primer design in addition with the equine *ASIP* sequence (AF288358).

The DNA of 4 individuals representing the chestnut phenotype and 20 melanistic roe deer were amplified in a volume of 35 mL containing 50 ng DNA, 0.2 μM of each primer, 0.2 mM dNTP, 2.5 mM MgCl_2_, and 1 U GoTaqflexi polymerase (Promega, Madison, WI, USA). This was followed by PCR which included a denaturation step at 95 °C (5 min) followed by 35 cycles of denaturation at 94 °C (60 s), annealing at primer specific temperature (30 s), and elongation at 72 °C (45 s). The final elongation step was carried out at 72 °C for 5 min. After running on a 2% agarose gel for 30 min, the PCR fragments were cut out of the agarose gel and cleaned with a GeneJet Gel Extraction Kit (Fermentas, Thermo Fisher Scientific, Waltham, MA, USA). The sequencing was carried out as a Sanger reaction with a BigDye Terminator v1.1 Ready Reaction Cycle Sequencing Kit in an ABI PRISM 310 Genetic Analyzer (Life Technologies, Thermo Fisher Scientific, Waltham, MA, USA) applying the manufacturer’s protocol.

### 2.3. Calculation of Amino Acid Substitution Effect

The possible effects of amino acid substitution were calculated by comparing 44 *ASIP* sequences from GenBank with SIFT (https://sift.bii.a-star.edu.sg/www/SIFT_seq_submit2.html) using standard settings.

### 2.4. Test of Target Mutations

The identification of causative mutations for specific phenotypes is often a challenge in wild animals. In domestic animals and model species, pedigree data are available and crossbreeding is a possible tool for identification of underlying genetics, but other strategies have to be utilized for wild animals. We utilized a test approach for evaluation of the black mutation in European roe deer. Samples of 317 specimens from North-West Germany, 127 from United Kingdom and 27 from Saxony-Anhalt were included in a blind test scenario. A protocol was drawn up for each European roe deer shot, in which the coat color was recorded in addition to the specimen’s location. This phenotypic information was available after genotyping. Our approach was methodically adopted to the specifics of investigations of wild animals. In the end, the association between genotypes and phenotypes was statistically validated for the candidate mutations. Genotyping of the target *ASIP*-SNP (c.33G>T) was done with KBiosciences Allele Specific PCR (KASP) technology using standard procedure and a 57 °C touch-down PCR approach (LGC Genomics, Berlin, Germany). Primer sequences, fragment lengths, and annealing temperatures are listed in Table 1 below.

### 2.5. Statistical Analysis

After identifying a candidate gene mutation for melanism, we tested the genotype counts for Hardy–Weinberg equilibrium. Then, we tested if genotype–phenotype combinations significantly departed from randomness by means of contingency-table tests. Finally, to test specifically if the candidate genotype (TT) was the cause of melanism, we applied a custom test that consisted of calculating the one-tail probability of observing less TT genotypes in non-melanistic (chestnut) individuals than in the observed empirical sample. The rationale is that if TT is a causative variant of melanism there will be an excess of individuals with both TT genotypes and melanism than would be expected by chance. This is the same as measuring the deficit of non-melanistic individuals with TT genotype, which is easier to compute. The obtained probability can be interpreted as a *p*-value of the one-tail test of TT being overrepresented in individuals with melanism.

For this reason, we employed a multinomial distribution with categories of phenotype–genotype combinations, and unknown population frequencies were approached by sampling frequencies. We replicated the analysis to discard the effect of different subjective choices. For instance: Should the population genotype categories be equal to the observed proportions or should they be calculated under Hardy–Weinberg equilibrium? Because of this, we applied both the multinomial test and the contingency tables for the following test categories: two genotype categories, three genotype categories, allele frequencies (rather than genotypes), with and without Hardy–Weinberg equilibrium, and for each geographical sample, and with all samples pooled together.

## 3. Results

The sequences were archived in GenBank (MT518869-72). Altogether 1384 bp of the *MC1R* gene were sequenced successfully. In agreement with other species, the *MC1R* of European roe deer has a single exon of 954 bp (318 amino acids) (Figure S1). In comparison with the draft genome, we found only one substitution at position c.444C>T. Three animals (1 chestnut and 2 black) were heterozygous CT of this SNP, while the others (1 chestnut and 3 black) had the same homozygous genotype CC as also found in the draft genome of European roe deer as well as in the GenBank European roe deer sequence. However, this is a synonymous mutation p.Tyr148Tyr without amino acid exchange.

We sequenced the largest part of the *ASIP* gene (2039 bp) (Figure S2). Largely, 225 bp containing 75 amino acids of the exome were examined. Only a part of the second intron and the exon 3 were missing in our analysis. Four SNPs were detected in the *ASIP* gene. For the evaluation of these SNPs, the sample set was extended by 2 chestnut and 15 black animals. Coat colors and SNP distribution of these specimens are shown in Table 2. The SNP c.33G>T located in exon 1 has the strongest association with the black coat color.

### 3.1. Amino Acid Substituion

**Figure 2:**
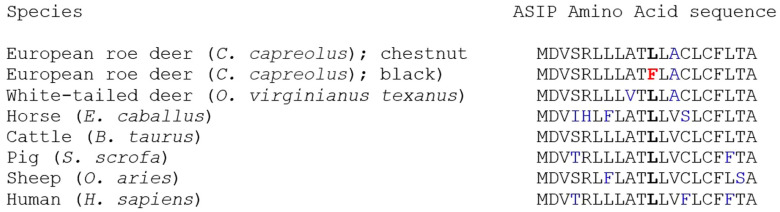
Alignment of amino acid sequences for the first part of *ASIP* exon 1 (AS1 to AS20) for white-tailed deer (GenBank XM_020889165), horse (GenBank AF288358), cattle (GenBank AH013175), pig (GenBank GQ373180), sheep (GenBank EU420022), and human (GenBank NM_001672). AA p.11 is bold, change in melanistic European roe deer is red, interspecific amino acids are blue.

The SNP c.33G>T leads to an amino acid substitution p.Leu11Phe. The leucine variant can also be found in this position in the *ASIP* gene in numerous other species, while the phenylalanine variant only occurs in the mutant allele of the black roe deer (Figure 2).

The calculation of the possible effects of the amino acid substitution with SIFT demonstrated that the exchange is rather to be regarded as intolerant with a score of 0.00. However, there is low confidence in this prediction because the sequences are not diverse enough.

### 3.2. Result of the Genetic Test

The most promising SNP in exon 1 of *ASIP* was targeted in a blind test. A total of 471 European roe deer from different regions in Germany and United Kingdom were genotyped with an Allele Specific PCR (KASP) for the SNP c.33G>T (Table 3). In the post-genotype comparison with the coat color phenotypes, it was found that all melanistic animals were homozygous TT at position c.33, while the chestnut individuals were either homozygous GG or heterozygous GT but never homozygous TT.

The observed associations were nonrandom in the pooled sample as well as for North-West Germany, and Saxony-Anhalt, Germany, while non-computable for the United Kingdom sample because it lacked melanistic individuals in general (Table 4). The total absence of TT genotypes in chestnut specimens produced exceedingly low probabilities in the contingency tests and astronomically low probabilities in the multinomial test. As expected, local samples, except from the United Kingdom, had higher values than the pooled ones, especially Saxony-Anhalt, Germany, due to their lower sample size. In general, the complete association of TT genotypes and melanistic individuals with the large sample sizes (almost 500 in the pooled sample) provides strikingly high statistical support to the hypothesis of the TT genotype causing melanism.

The genotype frequencies were also strong in Hardy–Weinberg disequilibrium in North-West Germany (*p*-value < 1.0 × 10^−8^), Saxony-Anhalt, Germany (*p*-value = 1.4 × 10^−6^), and pooled (*p*-value < 1.0 × 10^−8^), but not in the United Kingdom samples (*p*-value = 0.999).

## 4. Discussion

The MC1R has an important role in the alternative activation of the metabolic pathway of production of the pigments eumelanin (blackish) and pheomelanin (reddish). As a result, all carriers of a dominant black allele of *MC1R* will have black fur. The black jaguar is a well-known example of a dominant black *MC1R* mutation [1]. However, the *MC1R*-SNP c.444C>T found in European roe deer is a synonymous mutation and does not segregate in both phenotypes. Therefore, we exclude the *MC1R* as the reason for blackening in European roe deer.

In many species, the coat color arises, apart from possible lightening and spotting [3], through the interaction of the MC1R with the antagonistic ASIP. In contrast to the systemic active MC1R, ASIP acts at a local scale only. Due to the local expression of *ASIP*, the genetic ability to produce black eumelanin is reduced to certain areas of the body. We observed four SNPs in *ASIP* (Table 2), but only the SNP c.33G>T segregated both phenotypes. All black-colored European roe deer were homozygous TT at this SNP. This outcome was confirmed by our blind test of 471 specimens in which the black phenotype was assigned after genotyping all homozygous TT animals (*n* = 13), while chestnut specimens shared either the homozygote GG genotype (*n* = 432) or the heterozygote GT genotype (*n* = 26) (Table 2). With the addition of these specimens to the originally tested 24 specimens (TT = 20; 4 = GG), this sample set provided a respectable statistical power that resulted in extremely low probabilities of being caused by sampling error alone (Table 4). Values were still highly significant for each geographical location as long as the tests were possible. The high significance was also unaffected by considering Hardy–Weinberg equilibrium or different genotype systems, strongly suggesting that the melanistic phenotype has no plasticity or epigenetic components and is rather caused by a single substitution.

The substitution of G by T causes an amino acid exchange (p.Leu11Phe) which produces evidence for intolerance of protein function. Notably, white-tailed deer (XM_020889165), horses [11], wild cattle [12], pig [13], sheep [14], and human [15] also have the leucine variant at the amino acid position mentioned (Figure 2), indicating its basal origin. It is likely that leucine is the non-mutated, primitive character of this triplet, which seems to allow ASIP to suppress the blackish eumelanin by blocking the MC1R, resulting in the common phenotype of chestnut-colored European roe deer as reported for many other species. Chestnut-colored European roe deer can have an active ASIP suppressing the blackish eumelanin on nearly their entire body (Figure 1).

Chestnut-colored European roe deer have at least one non-mutated allele. In contrast, all black animals had two mutated alleles, which produced a strong association of the black color and the reported SNP. This would result in a recessive mode of inheritance of the mutated *ASIP* allele, which is also found in leopards, horses, and other animals [1,11,16].

Altogether and regardless of other genetic reasons for melanism, e.g., [17,18,19,20,21,22,23], investigation of segregation of both alleles, mutated and non-mutated, in a set of 495 samples produced a perfect association resulting in extremely high statistical support under different scenarios and providing strong arguments that the mutation c.33.G>T causes the black phenotype in European roe deer. Considering the high frequency of melanistic individuals in the North-West German lowland population, it cannot be excluded that melanism had a selective advantage (e.g., camouflage) as previously discussed for other species [3,18,24]. In addition, artificial selection based on human preferences (e.g., hunting restrictions) is a likely reason for its frequent occurrence. Although this study focused on two important genes for melanism in European roe deer, a genome-wide association study and expression analysis should be done to give information about the possibility of further associated genes to the black coat color in European roe deer.

## Figures and Tables

**Table 1:** Primers and annealing temperatures.

Primer	Sequence 5′–3′	Length	Temp.
MC1R-1 up	CCC ACG GGC CAG GAG GAA	708 bp	62 °C
MC1R-1 low	GCA GGG CGT AGA AGA TGG AGA TGT		
MC1R-2 up	GCC ATC GCC AAG AAC CGC AAC C	944 bp	63 °C
MC1R-2 low	ACC ATC TCC CCA GCC TCC TCA TTC		
ASIP-A up	GGC ATT ACT GGG GAC CTA TCA AC	941 bp	56 °C
ASIP-A low	CAA CCC TGG CAT GAA AGA ACT A		
ASIP-B up	CCC CAA GCC GCT ATC AGG A	1068 bp	56 °C
ASIP-B low	TGC AGA CCT AGA GCC AGA GAC		
ASIP-C up	GGG ATA CCG GAA ACA CAA GAC CAT	469 bp	56 °C
ASIP-C low	GGC ATG CAA CCC TGG ACA ATC		
ASIP-33-A1	GAA GCA CAG GCA GGC CAG C	44 bp	57 °C
ASIP-33-A2	GGA AGC ACA GGC AGG CCA GA		
ASIP-33-C	CAG CCG CCT CCT CCT GGC TA		

**Table 2:** Distribution of the four SNPs in the *ASIP* gene depending on coat color.

SNP	Region	Genotype	Black	Chestnut
c.1-270C>A	Promotor	CC	19	4
		CA	1	0
c.1-91A>G	Promotor	AA	14	4
		AG	6	0
c.33G>T	Exon 1	GG	0	4
		TT	20	0
c.161-170C>A	Intron 1	CC	20	2
		CA	0	2

**Table 3:** Results of blind test of SNP c.33G>T in 471 European roe deer (details in Table S1).

Origin	GG	GT	TT	Phenotype
North-West Germany	281			Chestnut
		25		Chestnut
			11	Black
United Kingdom	126			Chestnut
		1		Chestnut
Saxony-Anhalt Germany	25			Chestnut
			2	Black

**Table 4:** Tests of randomness of genotype and phenotype frequencies. Multinomial and contingency table tests were carried out for different types of categories for genotypes, for each geographical sample, and with two or three genotype categories. As we tested associations with two phenotypes hypothesized to be caused by two genotype categories, a two genotype categories test was statistically justified. NA means that the test was not able to be computed, generally because of mathematical obstacles (e.g., a division by zero).

	Type of Genotype Categories	Nr. of Genotype Categories	Sample	χ^2^	*p*-Value
Contingency table	Independent genotypes	2: (GG/GT), (TT)	North-West Germany	341.00	<1.0 × 10^−10^
			United Kingdom	NA *	NA *
			Saxony-Anhalt Germany	27.000	2.03 × 10^−7^
			Pooled	495.00	<1.0 × 10^−10^
		3: (GG), (GT), (TT)	North-West Germany	341.00	<1.0 × 10^−10^
			United Kingdom	NA *	NA *
			Saxony-Anhalt Germany	27.000	1.37 × 10^−6^
			Pooled	495.00	<1.0 × 10^−10^
	Hardy–Weinberg equilibrium	2: (GG/GT), (TT)	North-West Germany	1879.1	<1.0 × 10^−10^
			United Kingdom	0.00196 **	0.9646 **
			Saxony-Anhalt Germany	362.64	<1.0 × 10^−10^
			Pooled	3792.3	<1.0 × 10^−10^
		3: (GG), (GT), (TT)	North-West Germany	1917.3	<1.0 × 10^−10^
			United Kingdom	0.00198 **	0.999 **
			Saxony-Anhalt Germany	366.66	<1.0 × 10^−10^
			Pooled	3835.0	<1.0 × 10^−10^
	By alleles	Alleles: (G), (T)	North-West Germany	466.43	<1.0 × 10^−10^
			United Kingdom	NA *	NA *
			Saxony-Anhalt Germany	54.000	<1.0 × 10^−10^
			Pooled	690.23	<1.0 × 10^−10^
Multinomial	Independent genotypes	2: (GG/GT), (TT)	North-West Germany		5.75 × 10^−47^
			United Kingdom		NA *
			Saxony-Anhalt Germany		0.00022
			Pooled		1.59 × 10^−54^
		3: (GG), (GT), (TT)	North-West Germany		4.77 × 10^−48^
			United Kingdom		NA ***
			Saxony-Anhalt Germany		NA ***
			Pooled		2.22 × 10^−56^
	Hardy–Weinberg equilibrium	2: (GG/GT), (TT)	North-West Germany		1.66 × 10^−59^
			United Kingdom		NA ***
			Saxony-Anhalt Germany		7.37 × 10^−6^
			Pooled		1.19 × 10^−84^
		3: (GG), (GT), (TT)	North-West Germany		9.92 × 10^−71^
			United Kingdom		NA ***
			Saxony-Anhalt Germany		1.81 × 10^−7^
			Pooled		1.03 × 10^−71^
	By alleles	Alleles: (G), (T)	North-West Germany		1.16 × 10^−70^
			United Kingdom		NA ***
			Saxony-Anhalt Germany		1.30 × 10^−7^
			Pooled		1.21 × 10^−84^

* Not computable, too many zeros. ** Dubious validity, too many missing categories. *** Not computable, undetermined value.

## Supplementary Materials

The following are available online at https://www.mdpi.com/2073-4425/11/6/647/s1, Figure S1: Alignment of *MC1R* based on European roe deer sequences from the whole genome sequencing, the GenBank sequence, the chestnut and the black roe deer. Figure S2: Alignment of *ASIP* based on sequences of the European roe deer from the whole genome sequencing, a chestnut and a melanistic individual, and the equine mRNA sequence. Table S1: Table of origin, coat color phenotype and genotype for SNPs for 495 European roe deer.
