# Supplementary material for: An Agouti-Signaling-Protein Mutation is Strongly Associated with Melanism in European Roe Deer (Capreolus capreolus)
Source: Genes (Basel). 2020 Jun 11;11(6):647. doi: 10.3390/genes11060647 (PMC7349051; doi:10.3390/genes11060647)
Supplement: Supplementary file 1 [file genes-11-00647-s001.pdf]

Supplementary Material

Figure S1. Alignment (1384 bp) of *MC1R* based on European roe deer sequences from the whole genome sequencing (>lcl|Deer_k43_scaffold143768:28399-31352 10.1: green), the GenBank sequence (Y13960: blue), the chestnut (brown) and the black roe deer (black), ATG and SNP c.444C>T bold.

CCCACGG GCCAGGAGGA

CCCACGG GCCAGGAGGA
CCCACGG GCCAGGAGGA

AGCAAGCGGC CCAGAAATGT CTGCCTGTGG GCAACCGCAC CTCCAGGGAA GAGGCGGGGA

AGCAAGCGGC CCAGAAATGT CTGCCTGTGG GCAACCGCAC CTCCAGGGAA GAGGCGGGGA
AGCAAGCGGC CCAGAAATGT CTGCCTGTGG GCAACCGCAC CTCCAGGGAA GAGGCGGGGA

CGCAGACTGA GGGCAGAAGA GCGACGCTGC ACCCAGAGGG CTGGCCCCAC GAGCTTGGGG

CGCAGACTGA GGGCAGAAGA GCGACGCTGC ACCCAGAGGG CTGGCCCCAC GAGCTTGGGG
CGCAGACTGA GGGCAGAAGA GCGACGCTGC ACCCAGAGGG CTGGCCCCAC GAGCTTGGGG

GCCATGCCTG GGCCAACATT TGTCCAGCCA GGGAGGGGGG GGTGTGAGGC CCCTCCCAGG

GCCATGCCTG GGCCAACATT TGTCCAGCCA GGGAGGGGGG GGTGTGAGGC CCCTCCCAGG
GCCATGCCTG GGCCAACATT TGTCCAGCCA GGGAGGGGGG GGTGTGAGGC CCCTCCCAGG

GGAGCCATGG GTTGAGCAGG ACCCCGAGAG CAAGCAACCC TTCCTGCTCC CTGCGGGATG

GGAGCCATGG GTTGAGCAGG ACCCCGAGAG CAAGCAACCC TTCCTGCTCC CTGCGGGATG
GGAGCCATGG GTTGAGCAGG ACCCCGAGAG CAAGCAACCC TTCCTGCTCC CTGCGGGATG

c.1 **ATG**CCTGTGC TTGGCTCTCA GAGGCGGCTG CTGGGTTCCC TTAACTGCAC GCCTCCAGCC
c.1 **atg**cctgtgc ttggctctca gaggcggctg ctgggttccc ttaactgcac gcctccagcc
c.1 **ATG**CCTGTGC TTGGCTCTCA GAGGCGGCTG CTGGGTTCCC TTAACTGCAC GCCTCCAGCC
c.1 **ATG**CCTGTGC TTGGCTCTCA GAGGCGGCTG CTGGGTTCCC TTAACTGCAC GCCTCCAGCC

c.61 ACCTTCCCCC TCACGCTGGC CCCCAATCGG ACGGGGCCCC AGTGCCTGGA GGTGTCCATC
c.61 accttccccc tcacgctggc ccccaatcgg acggggcccc agtgcctgga ggtgtccatc
c.61 ACCTTCCCCC TCACGCTGGC CCCCAATCGG ACGGGGCCCC AGTGCCTGGA GGTGTCCATC
c.61 ACCTTCCCCC TCACGCTGGC CCCCAATCGG ACGGGGCCCC AGTGCCTGGA GGTGTCCATC

c.121 CCCGACGGGC TCTTTCTCAG CCTGGGGCTA GTGAGTCTCG TGGAGAATGT GCTGGTGGTG
c.121 cccgacgggc tctttctcag cctggggcta gtgagtctcg tggagaatgt gctggtggtg

```
c.121 CCCGACGGGC TCTTTCTCAG CCTGGGGCTA GTGAGTCTCG TGGAGAATGT GCTGGTGGTG
c.121 CCCGACGGGC TCTTTCTCAG CCTGGGGCTA GTGAGTCTCG TGGAGAATGT GCTGGTGGTG

c.181 GCTGCCATCG CCAAGAACCG CAACCTGCAC TCCCCCATGT ACTACTTCAT CTGCTGCCTG
c.181 gctgccatcg ccaagaaccg caacctgcac tcccccatgt actacttcat ctgctgcctg
c.181 GCTGCCATCG CCAAGAACCG CAACCTGCAC TCCCCCATGT ACTACTTCAT CTGCTGCCTG
c.181 GCTGCCATCG CCAAGAACCG CAACCTGCAC TCCCCCATGT ACTACTTCAT CTGCTGCCTG

c.241 GCTGTGTCCG ACCTGCTGGT AAGCGTCAGC AATGTGCTGG AGACAGCGGT CATGCTGCTG
c.241 gctgtgtccg acctgctggt aagcgtcagc aatgtgctgg agacagcggt catgctgctg
c.241 GCTGTGTCCG ACCTGCTGGT AAGCGTCAGC AATGTGCTGG AGACAGCGGT CATGCTGCTG
c.241 GCTGTGTCCG ACCTGCTGGT AAGCGTCAGC AATGTGCTGG AGACAGCGGT CATGCTGCTG

c.301 CTGGAGGCCG GTGCCCTGGC CGCCCGGGCA GCTGTGGTGC AGCAGCTGGA CAATGTCATC
c.301 ctggaggccg gtgccctggc cgcccgggca gctgtggtgc agcagctgga caatgtcatc
c.301 CTGGAGGCCG GTGCCCTGGC CGCCCGGGCA GCTGTGGTGC AGCAGCTGGA CAATGTCATC
c.301 CTGGAGGCCG GTGCCCTGGC CGCCCGGGCA GCTGTGGTGC AGCAGCTGGA CAATGTCATC

c.361 GACATGCTTA TCTGTGGCTC CATGGTATCC AGCCTCTGCT TCCTGGGCGC CATCGCTGTG
c.361 gacatgctta tctgtggctc catggtatcc agcctctgct tcctgggcgc catcgctgtg
c.361 GACATGCTTA TCTGTGGCTC CATGGTATCC AGCCTCTGCT TCCTGGGCGC CATCGCTGTG
c.361 GACATGCTTA TCTGTGGCTC CATGGTATCC AGCCTCTGCT TCCTGGGCGC CATCGCTGTG

c.421 GACCGCTACA TCTCCATCTT CTACGCCCTG CGGTACCACA GTGTCGTGAC ACTGCCCCGG
c.421 gaccgctaca tctccatctt ctacgccctg cggtaccaca gtgtcgtgac actgccccgg
c.421 GACCGCTACA TCTCCATCTT CTAYGCCCTG CGGTACCACA GTGTCGTGAC ACTGCCCCGG
c.421 GACCGCTACA TCTCCATCTT CTAYGCCCTG CGGTACCACA GTGTCGTGAC ACTGCCCCGG

c.481 GCGTGGCGGA TCATTGCGGC CATCTGGGTG GCCAGCATCC TCACCAGCCT TCTCTTCATC
c.481 gcgtggcgga tcattgcggc catctgggtg gccagcatcc tcaccagcct tctcttcatc
c.481 GCGTGGCGGA TCATTGCGGC CATCTGGGTG GCCAGCATCC TCACCAGCCT TCTCTTCATC
c.481 GCGTGGCGGA TCATTGCGGC CATCTGGGTG GCCAGCATCC TCACCAGCCT TCTCTTCATC

c.541 ACCTACTACA ACCACACGGT CGTCCTGCTG TGTCTCGTTG GCTTCTTCAT AGCCATGCTG
c.541 acctactaca accacacggt cgtcctgctg tgtctcgttg gcttcttcat agccatgctg
c.541 ACCTACTACA ACCACACGGT CGTCCTGCTG TGTCTCGTTG GCTTCTTCAT AGCCATGCTG
c.541 ACCTACTACA ACCACACGGT CGTCCTGCTG TGTCTCGTTG GCTTCTTCAT AGCCATGCTG

c.601 GCCCTCATGG CCGTCCTCTA CGTCCATATG CTGGCCCGGG CATGCCAGCA TGCCCGGGGC
c.601 gccctcatgg ccgtcctcta cgtccatatg ctggcccggg catgccagca tgcccggggc
c.601 GCCCTCATGG CCGTCCTCTA CGTCCATATG CTGGCCCGGG CATGCCAGCA TGCCCGGGGC
c.601 GCCCTCATGG CCGTCCTCTA CGTCCATATG CTGGCCCGGG CATGCCAGCA TGCCCGGGGC
```

c.661 ATCGCCCGGC TCCAGAAGAG GCAGCGTCCC ATCCATCAGG GCTTTGGCCT CAAGGGCGCT
c.661 atcgcccggc tccagaagag gcagcgtccc atccatcagg gctttggcct caagggcgct
c.661 ATCGCCCGGC TCCAGAAGAG GCAGCGTCCC ATCCATCAGG GCTTTGGCCT CAAGGGCGCT
c.661 ATCGCCCGGC TCCAGAAGAG GCAGCGTCCC ATCCATCAGG GCTTTGGCCT CAAGGGCGCT

c.721 GCCACCCTCA CCATCCTGCT GGGCGTCTTT TTTCTCTGCT GGGGCCCCTT CTTCCTGCAC
c.721 gccaccctca ccatcctgct gggcgtcttt tttctctgct ggggcccctt cttcctgcac
c.721 GCCACCCTCA CCATCCTGCT GGGCGTCTTT TTTCTCTGCT GGGGCCCCTT CTTCCTGCAC
c.721 GCCACCCTCA CCATCCTGCT GGGCGTCTTT TTTCTCTGCT GGGGCCCCTT CTTCCTGCAC

c.781 CTCTCGCTCA TCGTCCTCTG CCCCCAGCAT CCTACCTGTG GCTGCATCTT CAAGAACTTC
c.781 ctctcactca tcgtcctctg cccccagcat cctacctgtg gctgcatctt caagaacttc
c.781 CTCTCGCTCA TCGTCCTCTG CCCCCAGCAT CCTACCTGTG GCTGCATCTT CAAGAACTTC
c.781 CTCTCGCTCA TCGTCCTCTG CCCCCAGCAT CCTACCTGTG GCTGCATCTT CAAGAACTTC

c.841 AACCTCTTCC TGGCCCTCAT CATTTGCAAC GCCATCGTGG ACCCCCTCAT CTACGCCTTT
c.841 aacctcttcc tggccctcat catttgcaac gccatcgtgg accccctcat ctacgccttt
c.841 AACCTCTTCC TGGCCCTCAT CATTTGCAAC GCCATCGTGG ACCCCCTCAT CTACGCCTTT
c.841 AACCTCTTCC TGGCCCTCAT CATTTGCAAC GCCATCGTGG ACCCCCTCAT CTACGCCTTT

c.901 CGCAGCCAGG AGCTCCGGAA GACACTCCAA GAGGTGCTGC AGTGCTCCTG GTGAGGCTGG
c.901 cgcagccagg agctccggaa gacactccaa gaggtgctgc agtgctcctg gtga
c.901 CGCAGCCAGG AGCTCCGGAA GACACTCCAA GAGGTGCTGC AGTGCTCCTG GTGAGGCTGG
c.901 CGCAGCCAGG AGCTCCGGAA GACACTCCAA GAGGTGCTGC AGTGCTCCTG GTGAGGCTGG

CAGTGCCGTT GTGTGCCCCA GGCCTGTGAG GCCGGGGCAG TCCCTTGGCA AAGAGGATTG

CAGTGCCGTT GTGTGCCCCA GGCCTGTGAG GCCGGGGCAG TCCCTTGGCA AAGAGGATTG
CAGTGCCGTT GTGTGCCCCA GGCCTGTGAG GCCGGGGCAG TCCCTTGGCA AAGAGGATTG

GCTAGGCCAT CTCTGAAGGT GAGGGTGCAC AGGCCTTCAG GCGCCTGAGA AGGGAATTGC

GCTAGGCCAT CTCTGAAGGT GAGGGTGCAC AGGCCTTCAG GCGCCTGAGA AGGGAATTGC
GCTAGGCCAT CTCTGAAGGT GAGGGTGCAC AGGCCTTCAG GCGCCTGAGA AGGGAATTGC

AGGACTCTCC AGGAGGCTGT GCAGAATGAG GAGGCTGGGG AGATGGT

AGGACTCTCC AGGAGGCTGT GCAGAATGAG GAGGCTGGGG AGATGGT
AGGACTCTCC AGGAGGCTGT GCAGAATGAG GAGGCTGGGG AGATGGT

Figure S2. Alignment (2039 bp) of *ASIP* based on sequences of the European roe deer from the whole genome sequencing (>lcl|Deer_k43_scaffold320422:2380-9700 13.5: green), a chestnut (brown) and a melanistic individual (black), and the equine mRNA sequence (GenBank AF288358: blue). ATG and SNPs are bold.

```
                                                      GGCAT TACTGGGGAC CTATCAACAA
                                                      GGCAT TACTGGGGAC CTATCAACAA
                                                      GGCAT TACTGGGGAC CTATCAACAA

      TTCTGCTTAG GCCTTGGGTC TCCTGGAGCC ACTGGCTTAT AAAATGAAAC AAAAAGAACA
      TTCTGCTTAG GCCTTGGGTC TCCTGGAGCC ACTGGCTTAT AAAATGAAAC AAAAAGAACA
      TTCTGCTTAG GCCTTGGGTC TCCTGGAGCC ACTGGCTTAT AAAATGAAAC AAAAAGAACA

      CCCCACCCTC TGGAAGGCAG AGACCAATTG CAATTCATTC TTTTTCTCTT TGCAATCTCC
      CCCCACCCTC TGGAAGGCAG AGACCAATTG CAATTCATTC TTTTTCTCTT TGCAATCTCC
      CCCCACCCTC TGGAAGGCAG AGACCAATTG MAATTCATTC TTTTTCTCTT TGCAATCTCC

      CTCCCTTCCT TCCTTGTTTT TCTTCCTCTC CCCTCTCTCC TTTCCTCTCT TTTTCCTTCA
      CTCCCTTCCT TCCTTGTTTT TCTTCCTCTC CCCTCTCTCC TTTCCTCTCT TTTTCCTTCA
      CTCCCTTCCT TCCTTGTTTT TCTTCCTCTC CCCTCTCTCC TTTCCTCTCT TTTTCCTTCA

      TGAGTTCTCC TTCCCTGTCC TAAGCCTTGC TGGTCCTCCA GCTCCACTGG GACTCTGGGC
      TGAGTTCTCC TTCCCTGTCC TAAGCCTTGC TGGTCCTCCA GCTCCACTGG GACTCTGGGC
      TGAGTTCTCC TTCCCTGTCC TAAGCCTTGC TGGTCCTCCA GCTCCACTGG GACTCTGGGC

      TGTGGCCTGG GGGTCAGAGC ACCAGCCCAA AGAAACATAA AGAAAGCAGG AGGGCACACG
      TGTGGCCTGG GGGTCAGAGC ACCAGCCCAA AGAAACATAA AGAAAGCAGG AGGGCACACG
      TGTGGCCTGG GGGTCAGAGC ACCAGCCCAR AGAAACATAA AGAAAGCAGG AGGGCACACG

      CATTTGCCAG AACCCCCGGC CCACCTGACT GCCTTCTCTG TCCCTCTCAG GCCTCCTGGG
      CATTTGCCAG AACCCCCGGC CCACCTGACT GCCTTCTCTG TCCCTCTCAG GCCTCCTGGG
      CATTTGCCAG AACCCCCGGC CCACCTGACT GCCTTCTCTG TCCCTCTCAG GCCTCCTGGG

  c.1 ATGGACGTCA GCCGCCTCCT CCTGGCTACC TTGCTGGCCT GCCTGTGCTT CCTCACTGCC
  c.1 ATGGACGTCA GCCGCCTCCT CCTGGCTACC TTGCTGGCCT GCCTGTGCTT CCTCACTGCC
  c.1 ATGGACGTCA GCCGCCTCCT CCTGGCTACC TTTCTGGCCT GCCTGTGCTT CCTCACTGCC
  c.1 atggatgtca ttcacctgtt cctggccacc ctgctggtca gcctctgctt cctcactgcc

 c.61 TACAGCCACC TGGCACCTGA GGAAAAGCCC AGAGATGAAA GGAACCTGAA GAACAACTCT
 c.61 TACAGCCACC TGGCACCTGA GGAAAAGCCC AGAGATGAAA GGAACCTGAA GAACAACTCT
 c.61 TACAGCCACC TGGCACCTGA GGAAAAGCCC AGAGATGAAA GGAACCTGAA GAACAACTCT
 c.61 tacagccacc tgtcacctga ggagaagccc aaagatgaca ggagcctgag gaacaactcc

c.121 TCCATGAACC TGTTAGATTT CCCTTCTGTC TCTATTGTGG GTAAGTAGTC TTACCTACTG
c.121 TCCATGAACC TGTTAGATTT CCCTTCTGTC TCTATTGTGG GTAAGTAGTC TTACCTACTG
```

c.121 TCCATGAACC TGTTAGATTT CCCTTCTGTC TCTATTGTGG GTAAGTAGTC TTACCTACTG
c.121 tccatgaacc tgttggattc cccttctgtc tctatcatgg

GCTGGCCTGG GGCCCAGCCT CTGGGCTCTG GCCCATGAGA AGGGGCTGAA GGGGGTCAAA
GCTGGCCTGG GGCCCAGCCT CTGGGCTCTG GCCCATGAGA AGGGGCTGAA GGGGGTCAAA
GCTGGCCTGG GGCCCAGCCT CTGGGCTCTG GCCCATGAGA AGGGGCTGAA GGGGGTCAAA

CACTCCCCAA GCCGCTATCA GGATCCATTG CCATTGGTGA GAGCTCCTTT GTGCTCATTC
CACTCCCCAA GCCGCTATCA GGATCCATTG CCATTGGTGA GAGCTCCTTT GTGCTCATTC
CACTCCCCAA GCCGCTATCA GGATCCATTG CCATTGGTGA GAGCTCCTTT GTGCTCATTC

CTCAGAACAA TTCTGGAGGA ATCAAGTGCC CCTTAACTCA TTTAGTTAAG AAAGCTCTGC
CTCAGAACAA TTCTGGAGGA ATCAAGTGCC CCTTAACTCA TTTAGTTAAG AAAGCTCTGC
CTCAGAACAA TTCTGGAGGA ATCAAGTGCC CCTTAACTCA TTTAGTTAAG AAAGCTCTGC

CTCTCACCAA TCTAATATGC AGCTTAGCAT GTTAGAGGCT CTGAGAAACC CTAAAGTCAA
CTCTCACCAA TCTAATATGC AGCTTAGCAT GTTAGAGGCT CTGAGAAACC CTAAAGTCAA
CTCTCACCAA TCTAATATGC AGCTTAGCAT GTTAGAGGCT CTGAGAAACC CTAAAGTCAA

GACACCTGGT GAATTTGGTT TAACTTGGTG TTTCTGAAAC TGAATCACGT AACTCCTATC
GACACCTGGT GAATTTGGTT TAACTTGGTG TTTCTGAAAC TGAATCACGT AACTCCTATC
GACACCTGGT GAATTTGGTT TAACTTGGTG TTTCTGAAAC TGAATCACGT AACTCCTATC

AATGGTAAAA TTTTTAGAAA ATTCTGCAGA ATATAACCAA GTCTGGGATG AACTAGTTCT
AATGGTAAAA TTTTTAGAAA ATTCTGCAGA ATATAACCAA GTCTGGGATG AACTAGTTCT
AATGGTAAAA TTTTTAGAAA ATTCTGCAGA ATATAACCAA GTCTGGGATG AACTAGTTCT

TTCATGCCAG GGTTGAGAAT TGAAAATGAC TGCTCAGAGG AAAATATCAG GGGTATTAGA
TTCATGCCAG GGTTGAGAAT TGAAAATGAC TGCTCAGAGG AAAATATCAG GGGTATTAGA
TTCATGCCAG GGTTGAGAAT TGAAAATGAC TGCTCAGAGG AAAATATCAG GGGTATTAGA

GCCTAGCATT AGAGAATTTC TGGTTCGAAT TCATCTGACA ATAATTATAA TAACATATAT
GCCTAGCATT AGAGAATTTC TGGTTCGAAT TCATCTGACA ATAATTATAA TAACATATAT
GCCTAGCATT AGAGAATTTC TGGTTCGAAT TCATCTGACA ATAATTATAA TAACATATAT

ACTTATTTTC TGATAGGCAC TCTGAAGTGA ATACTATTTT TATTCCTATT TCACAGCTAA
ACTTATTTTC TGATAGGCAC TCTGAAGTGA ATACTATTTT TATTCCTATT TCACAGCTAA
ACTTATTTTC TGATAGGCAC TCTGAAGTGA ATACTATTTT TATTCCTATT TCACAGCTAA

GTAAATTAAG AGTTAGAAAA GTTAAGTAAC TAACATACAG TTTGTAACTG GCAGAGACAG
GTAAATTAAG AGTTAGAAAA GTTAAGTAAC TAACATACAG TTTGTAACTG GCAGAGACAG
GTAAATTAAG AGTTAGAAAA GTTAAGTAAC TAACATACAG TTTGTAACTG GCAGAGACAG

TCCATAGGTC GCAAAGATTA AGACACGACT GAGCGACTGA ACTGAACTGA CTGAACTGAA
TCCATAGGTC GCAAAGATTA AGACACGACT GAGCGACTGA ACTGAACTGA CTGAACTGAA
TCCATAGGTC GCAAAGATTA AGACACGACT GAGCGACTGA ACTGAACTGA CTGAACTGAA

TACATACCCA GGTTCTGGGT TAGGTTCTGT TTAACTCCAG AATCTAAGCT CAAAACTACT
TACATACCCA GGTTCTGGGT TAGGTTCTGT TTAACTCCAG AATCTAAGCT CAAAACTACT
TACATACCCA GGTTCTGGGT TAGGTTCTGT TTAACTCCAG AATCTAAGCT CAAAACTACT

ATTCCTTACT GACTTGCCTC TCACTTCAAA AAGAGGATAT TCTAGATATA TGGGAAGGGA
ATTCCTTACT GACTTGCCTC TCACTTCAAA AAGAGGATAT TCTAGATATA TGGGAAGGGA
ATTCCTTACT GACTTGCCTC TCACTTCAAA AAGAGGATAT TCTAGATATA TGGGAAGGGA

CTGGAGCACA TTAACATTGA TCTGTGTCAG ATTTTCCACT ATATAAACAT ACCTCATTTA
CTGGAGCACA TTAACATTGA TCTGTGTCAG ATTTTCCACT ATATAAACAT ACCTCATTTA
CTGGAGCACA TTAACATTGA TCTGTGTCAG ATTTTCCACT ATATAAACAT ACCTCATTTA

TCCTCACAAC TCTTCAAGGT AGGTATTATA GTACCTACTT GCATATGAGA AAACTGAGTC
TCCTCACAAC TCTTCAAGGT AGGTATTATA GTACCTACTT GCATATGAGA AAACTGAGTC
TCCTCACAAC TCTTCAAGGT AGGTATTATA GTACCTACTT GCATATGAGA AAACTGAGTC

TTAAGGAGAT TAAGTAGGTT GTCCAAGACC ACAAAGCTGT TATGTAGCAG AGTCAGGATT
TTAAGGAGAT TAAGTAGGTT GTCCAAGACC ACAAAGCTGT TATGTAGCAG AGTCAGGATT
TTAAGGAGAT TAAGTAGGTT GTCCAAGACC ACAAAGCTGT TATGTAGCAG AGTCAGGATT

CAAATCAGGT CTGCCTGGAT CCAAAGCTCT GAGAAATAGG AAATAGGGAT ACCGGAAACA
CAAATCAGGT CTGCCTGGAT CCAAAGCTCT GAGAAATAGG AAATAGGGAT ACCGGAAACA
CAAATCAGGT CTGCCTGGAT CCAAAGCTCT GAGAAATAGG AAATAGGGAT ACCGGAAACA

CAAGACCATC CTTGTAGGAG ACACTGAGTC CATTTTCCAG GGCCGCCTTG GATTTTCCCT
CAAGACCATC CTTGTAGGAG ACACTGAGTC CATTTTCCAG GGCCGCCTTG GATTTTCCCT
CAAGACCATC CTTGTAGGAG ACACTGAGTC CATTTTCCAG GGCCGCCTTG GATTTTCCCT

TTCGCTTTCC TTCAGAGGAC TCTCTGCTCT CTGTCTCTGG CTCTAGGTCT G**C**AGCCAGGC
TTCGCTTTCC TTCAGAGGAC TCTCTGCTCT CTGTCTCTGG CTCTAGGTCT G**M**AGCCAGGC
TTCGCTTTCC TTCAGAGGAC TCTCTGCTCT CTGTCTCTGG CTCTAGGTCT G**C**AGCCAGGC

CACAGAAACT GCAGGCCTAA GTCCCAAGAT ATGATCTATC CAACCAAACC TTCATCCTCT
CACAGAAACT GCAGGCCTAA GTCCCAAGAT ATGATCTATC CAACCAAACC TTCATCCTCT
CACAGAAACT GCAGGCCTAA GTCCCAAGAT ATGATCTATC CAACCAAACC TTCATCCTCT

CCCCAACCCT GGGGCTTCCT AGAGCCCTCT CTGCTCCTCC CACTTCACTG CAGTTAAGGA
CCCCAACCCT GGGGCTTCCT AGAGCCCTCT CTGCTCCTCC CACTTCACTG CAGTTAAGGA
CCCCAACCCT GGGGCTTCCT AGAGCCCTCT CTGCTCCTCC CACTTCACTG CAGTTAAGGA

```
c.161 CCCTCAGAAA ATGGCTTTGT TCCTTCTGTC TCTCTTTGAA GCACTGAACA AGAAATCCAA
c.161 CCCTCAGAAA ATGGCTTTGT TCCTTCTGTC TCTCTTTGAA GCACTGAACA AGAAATCCAA
c.161 CCCTCAGAAA ATGGCTTTGT TCCTTCTGTC TCTCTTTGAA GCACTGAACA AGAAATCCAA
c.161                                              cattgaaca agaaatccaa

c.180 AAAGATCAGC AGAAATGAAG CTGAAAAGAA GAAAAGACCT TCCAAGGTAG GCCTGGGAGT
c.180 AAAGATCAGC AGAAATGAAG CTGAAAAGAA GAAAAGACCT TCCAAGGTAG GCCTGGGAGT
c.180 AAAGATCAGC AGAAATGAAG CTGAAAAGAA GAAAAGACCT TCCAAGGTAG GCCTGGGAGT
c.180 aaagatcagc agaaaagaag cagaaaagaa gaagagatct tccaag

      TCACATTGTC AGGATGGGAC TGGACTTAAA GGGGGAGGAC ACCCAAACTC TGGATAGGAA
      TCACATTGTC AGGATGGGAC TGGACTTAAA GGGGGAGGAC ACCCAAACTC TGGATAGGAA
      TCACATTGTC AGGATGGGAC TGGACTTAAA GGGGGAGGAC ACCCAAACTC TGGATAGGAA

      CTAAATGAAA GATTGTCCAG GGTTGCATGC CCCAGAGAAA CTGAAAGCTA CCAAAACCTT
      CTAAATGAAA GATTGTCCAG GGTTGCATGC C
      CTAAATGAAA GATTGTCCAG GGTTGCATGC C
```

Table S1. Table of origin, coat colour phenotype and genotype for SNPs (1x *MC1R* gene; 4x *ASIP*) for 495 European roe deer. Animals used for initial sequencing are grey.

Animal	Use	Gebiet	Farbe	MC1R c.444 C>T	ASIP c.1-270 C>A	ASIP c.1-91 A>G	ASIP c.33 G>T	ASIP c.161 C>A
V0031	Sequencing	North-West Germany	Black	CT	CC	AG	TT	CC
V0032	Sequencing	North-West Germany	Black	CC	CC	AA	TT	CC
V0033	Sequencing	North-West Germany	Black	CT	CA	AA	TT	CC
V0034	Sequencing	North-West Germany	Black		CC	AA	TT	CC
V0035	Sequencing	North-West Germany	black		CC	AA	TT	CC
V0041	Sequencing	North-West Germany	black		CC	AG	TT	CC
V0042	Sequencing	North-West Germany	black		CC	AA	TT	CC
V0043	Sequencing	North-West Germany	black		CC	AA	TT	CC
V0044	Sequencing	North-West Germany	black		CC	AG	TT	CC
V0045	Sequencing	North-West Germany	black		CC	AA	TT	CC
V0046	Sequencing	North-West Germany	black		CC	AA	TT	CC
V0047	Sequencing	North-West Germany	black		CC	AG	TT	CC
V0048	Sequencing	North-West Germany	black		CC	AA	TT	CC
V0049	Sequencing	North-West Germany	black		CC	AA	TT	CC
V0050	Sequencing	North-West Germany	black		CC	AG	TT	CC
V0051	Sequencing	North-West Germany	black		CC	AA	TT	CC
V0053	Sequencing	North-West Germany	black		CC	AA	TT	CC
V0061	Sequencing	North-West Germany	black		CC	AA	TT	CC
V0038	Sequencing	Saxony-Anhalt	black	CC	CC	AG	TT	CC
V0052	Sequencing	Saxony-Anhalt	black	CC	CC	AA	TT	CC
V0023	Sequencing	Tierpark Berlin	chestnut	CT	CC	AA	GG	CA
V0024	Sequencing	Tierpark Berlin	chestnut	CC	CC	AA	GG	CC
V0039	Sequencing	Tierpark Berlin	chestnut		CC	AA	GG	CC
V0040	Sequencing	Tierpark Berlin	chestnut		CC	AA	GG	CA
V0438	Blind test, KASP	United Kingdom	chestnut				GG	
V0439	Blind test, KASP	United Kingdom	chestnut				GG	
V0440	Blind test, KASP	United Kingdom	chestnut				GG	
V0441	Blind test, KASP	United Kingdom	chestnut				GG	
V0442	Blind test, KASP	United Kingdom	chestnut				GG	
V0443	Blind test, KASP	United Kingdom	chestnut				GG	
V0444	Blind test, KASP	United Kingdom	chestnut				GG	
V0445	Blind test, KASP	United Kingdom	chestnut				GG	
V0446	Blind test, KASP	United Kingdom	chestnut				GG	
V0447	Blind test, KASP	United Kingdom	chestnut				GG	
V0448	Blind test, KASP	United Kingdom	chestnut				GG	
V0449	Blind test, KASP	United Kingdom	chestnut				GG	
V0450	Blind test, KASP	United Kingdom	chestnut				GG	
V0451	Blind test, KASP	United Kingdom	chestnut				GG	

V0452	Blind test, KASP	United Kingdom	chestnut				GG	
V0453	Blind test, KASP	United Kingdom	chestnut				GG	
V0455	Blind test, KASP	United Kingdom	chestnut				GG	
V0456	Blind test, KASP	United Kingdom	chestnut				GG	
V0457	Blind test, KASP	United Kingdom	chestnut				GG	
V0458	Blind test, KASP	United Kingdom	chestnut				GG	
V0459	Blind test, KASP	United Kingdom	chestnut				GG	
V0460	Blind test, KASP	United Kingdom	chestnut				GG	
V0461	Blind test, KASP	United Kingdom	chestnut				GG	
V0462	Blind test, KASP	United Kingdom	chestnut				GG	
V0463	Blind test, KASP	United Kingdom	chestnut				GG	
V0464	Blind test, KASP	United Kingdom	chestnut				GG	
V0465	Blind test, KASP	United Kingdom	chestnut				GG	
V0466	Blind test, KASP	United Kingdom	chestnut				GG	
V0467	Blind test, KASP	United Kingdom	chestnut				GG	
V0468	Blind test, KASP	United Kingdom	chestnut				GG	
V0469	Blind test, KASP	United Kingdom	chestnut				GG	
V0470	Blind test, KASP	United Kingdom	chestnut				GG	
V0471	Blind test, KASP	United Kingdom	chestnut				GG	
V0472	Blind test, KASP	United Kingdom	chestnut				GG	
V0473	Blind test, KASP	United Kingdom	chestnut				GG	
V0474	Blind test, KASP	United Kingdom	chestnut				GG	
V0475	Blind test, KASP	United Kingdom	chestnut				GG	
V0476	Blind test, KASP	United Kingdom	chestnut				GG	
V0477	Blind test, KASP	United Kingdom	chestnut				GG	
V0478	Blind test, KASP	United Kingdom	chestnut				GG	
V0479	Blind test, KASP	United Kingdom	chestnut				GG	
V0480	Blind test, KASP	United Kingdom	chestnut				GG	
V0481	Blind test, KASP	United Kingdom	chestnut				GG	
V0482	Blind test, KASP	United Kingdom	chestnut				GG	
V0483	Blind test, KASP	United Kingdom	chestnut				GG	
V0484	Blind test, KASP	United Kingdom	chestnut				GG	
V0485	Blind test, KASP	United Kingdom	chestnut				GG	
V0486	Blind test, KASP	United Kingdom	chestnut				GG	
V0487	Blind test, KASP	United Kingdom	chestnut				GG	
V0488	Blind test, KASP	United Kingdom	chestnut				GG	
V0490	Blind test, KASP	United Kingdom	chestnut				GG	
V0492	Blind test, KASP	United Kingdom	chestnut				GG	
V0493	Blind test, KASP	United Kingdom	chestnut				GG	
V0494	Blind test, KASP	United Kingdom	chestnut				GG	
V0495	Blind test, KASP	United Kingdom	chestnut				GG	
V0496	Blind test, KASP	United Kingdom	chestnut				GG	
V0497	Blind test, KASP	United Kingdom	chestnut				GG	

V0499	Blind test, KASP	United Kingdom	chestnut				GG		
V0500	Blind test, KASP	United Kingdom	chestnut				GG		
V0501	Blind test, KASP	United Kingdom	chestnut				GG		
V0502	Blind test, KASP	United Kingdom	chestnut				GG		
V0503	Blind test, KASP	United Kingdom	chestnut				GG		
V0505	Blind test, KASP	United Kingdom	chestnut				GG		
V0506	Blind test, KASP	United Kingdom	chestnut				GG		
V0508	Blind test, KASP	United Kingdom	chestnut				GG		
V0509	Blind test, KASP	United Kingdom	chestnut				GG		
V0510	Blind test, KASP	United Kingdom	chestnut				GG		
V0511	Blind test, KASP	United Kingdom	chestnut				GG		
V0512	Blind test, KASP	United Kingdom	chestnut				GG		
V0513	Blind test, KASP	United Kingdom	chestnut				GG		
V0514	Blind test, KASP	United Kingdom	chestnut				GG		
V0515	Blind test, KASP	United Kingdom	chestnut				GG		
V0516	Blind test, KASP	United Kingdom	chestnut				GG		
V0517	Blind test, KASP	United Kingdom	chestnut				GG		
V0518	Blind test, KASP	United Kingdom	chestnut				GG		
V0519	Blind test, KASP	United Kingdom	chestnut				GG		
V0520	Blind test, KASP	United Kingdom	chestnut				GG		
V0521	Blind test, KASP	United Kingdom	chestnut				GG		
V0522	Blind test, KASP	United Kingdom	chestnut				GG		
V0524	Blind test, KASP	United Kingdom	chestnut				GG		
V0528	Blind test, KASP	United Kingdom	chestnut				GG		
V0530	Blind test, KASP	United Kingdom	chestnut				GG		
V0532	Blind test, KASP	United Kingdom	chestnut				GG		
V0534	Blind test, KASP	United Kingdom	chestnut				GG		
V0536	Blind test, KASP	United Kingdom	chestnut				GG		
V0538	Blind test, KASP	United Kingdom	chestnut				GG		
V0540	Blind test, KASP	United Kingdom	chestnut				GG		
V0542	Blind test, KASP	United Kingdom	chestnut				GG		
V0544	Blind test, KASP	United Kingdom	chestnut				GG		
V0546	Blind test, KASP	United Kingdom	chestnut				GG		
V0548	Blind test, KASP	United Kingdom	chestnut				GG		
V0550	Blind test, KASP	United Kingdom	chestnut				GG		
V0552	Blind test, KASP	United Kingdom	chestnut				GG		
V0554	Blind test, KASP	United Kingdom	chestnut				GG		
V0556	Blind test, KASP	United Kingdom	chestnut				GG		
V0558	Blind test, KASP	United Kingdom	chestnut				GG		
V0560	Blind test, KASP	United Kingdom	chestnut				GG		
V0562	Blind test, KASP	United Kingdom	chestnut				GG		
V0563	Blind test, KASP	United Kingdom	chestnut				GG		
V0564	Blind test, KASP	United Kingdom	chestnut				GG		

V0565	Blind test, KASP	United Kingdom	chestnut				GG	
V0566	Blind test, KASP	United Kingdom	chestnut				GG	
V0567	Blind test, KASP	United Kingdom	chestnut				GG	
V0568	Blind test, KASP	United Kingdom	chestnut				GG	
V0569	Blind test, KASP	United Kingdom	chestnut				GG	
V0570	Blind test, KASP	United Kingdom	chestnut				GG	
V0571	Blind test, KASP	United Kingdom	chestnut				GG	
V0572	Blind test, KASP	United Kingdom	chestnut				GG	
V0574	Blind test, KASP	United Kingdom	chestnut				GG	
V0575	Blind test, KASP	United Kingdom	chestnut				GG	
V0576	Blind test, KASP	United Kingdom	chestnut				GG	
V0577	Blind test, KASP	United Kingdom	chestnut				GG	
V0578	Blind test, KASP	United Kingdom	chestnut				GG	
V0579	Blind test, KASP	United Kingdom	chestnut				GG	
V0581	Blind test, KASP	United Kingdom	chestnut				GG	
V0582	Blind test, KASP	United Kingdom	chestnut				GG	
V0583	Blind test, KASP	United Kingdom	chestnut				GG	
V0584	Blind test, KASP	United Kingdom	chestnut				GG	
V0585	Blind test, KASP	United Kingdom	chestnut				GG	
V0586	Blind test, KASP	United Kingdom	chestnut				GG	
V0587	Blind test, KASP	United Kingdom	chestnut				GG	
V0588	Blind test, KASP	United Kingdom	chestnut				GT	
V0589	Blind test, KASP	United Kingdom	chestnut				GG	
V0590	Blind test, KASP	United Kingdom	chestnut				GG	
V0591	Blind test, KASP	United Kingdom	chestnut				GG	
V0592	Blind test, KASP	United Kingdom	chestnut				GG	
V0593	Blind test, KASP	United Kingdom	chestnut				GG	
BH-378	Blind test, KASP	North-West Germany	chestnut				GG	
BH-402A	Blind test, KASP	North-West Germany	chestnut				GG	
BH-402B	Blind test, KASP	North-West Germany	chestnut				GG	
BH-402C	Blind test, KASP	North-West Germany	chestnut				GG	
BH-413A	Blind test, KASP	North-West Germany	chestnut				GG	
BH-413B	Blind test, KASP	North-West Germany	chestnut				GG	
BH-413C	Blind test, KASP	North-West Germany	chestnut				GG	
BH-423	Blind test, KASP	North-West Germany	chestnut				GG	
BH-428	Blind test, KASP	North-West Germany	chestnut				GT	
BH-444	Blind test, KASP	North-West Germany	chestnut				GG	
BH-446	Blind test, KASP	North-West Germany	chestnut				GG	
BH-448	Blind test, KASP	North-West Germany	chestnut				GG	
BH-450	Blind test, KASP	North-West Germany	chestnut				GG	
BH-456A	Blind test, KASP	North-West Germany	chestnut				GG	
BH-456B	Blind test, KASP	North-West Germany	chestnut				GG	
BH-464	Blind test, KASP	North-West Germany	chestnut				GG	

BH-470A	Blind test, KASP	North-West Germany	chestnut				GG	
BH-470B	Blind test, KASP	North-West Germany	chestnut				GG	
BH-470C	Blind test, KASP	North-West Germany	chestnut				GG	
BH-472	Blind test, KASP	North-West Germany	chestnut				GG	
BH-508	Blind test, KASP	North-West Germany	chestnut				GG	
BH-509A	Blind test, KASP	North-West Germany	chestnut				GG	
BH-509B	Blind test, KASP	North-West Germany	chestnut				GG	
BH-509C	Blind test, KASP	North-West Germany	chestnut				GG	
BH-545	Blind test, KASP	North-West Germany	chestnut				GG	
Cc27	Blind test, KASP	North-West Germany	chestnut				GG	
Cc28	Blind test, KASP	North-West Germany	chestnut				GG	
Cc30	Blind test, KASP	North-West Germany	chestnut				GG	
Cc32	Blind test, KASP	North-West Germany	black				TT	
Cc34	Blind test, KASP	North-West Germany	chestnut				GG	
Cc35	Blind test, KASP	North-West Germany	chestnut				GG	
Cc36	Blind test, KASP	North-West Germany	chestnut				GT	
Cc37	Blind test, KASP	North-West Germany	chestnut				GT	
Cc38	Blind test, KASP	North-West Germany	chestnut				GG	
Cc39	Blind test, KASP	North-West Germany	chestnut				GG	
Cc42	Blind test, KASP	North-West Germany	black				TT	
Cc43	Blind test, KASP	North-West Germany	black				TT	
Cc44	Blind test, KASP	North-West Germany	chestnut				GG	
Cc45	Blind test, KASP	North-West Germany	chestnut				GG	
Cc46	Blind test, KASP	North-West Germany	chestnut				GG	
Cc47	Blind test, KASP	North-West Germany	black				TT	
Cc48	Blind test, KASP	North-West Germany	black				TT	
Cc49	Blind test, KASP	North-West Germany	chestnut				GG	
Cc50	Blind test, KASP	North-West Germany	black				TT	
Cc51	Blind test, KASP	North-West Germany	chestnut				GG	
Cc53	Blind test, KASP	North-West Germany	chestnut				GG	
Cc54	Blind test, KASP	North-West Germany	black				TT	
Cc55	Blind test, KASP	North-West Germany	black				TT	
Cc56	Blind test, KASP	North-West Germany	black				TT	
Cc58	Blind test, KASP	North-West Germany	chestnut				GG	
Cc59	Blind test, KASP	North-West Germany	chestnut				GG	
Cc60	Blind test, KASP	North-West Germany	chestnut				GG	
Cc61	Blind test, KASP	North-West Germany	chestnut				GG	
Cc62	Blind test, KASP	North-West Germany	chestnut				GG	
R-043	Blind test, KASP	North-West Germany	chestnut				GG	
R-044	Blind test, KASP	North-West Germany	chestnut				GG	
R-051	Blind test, KASP	North-West Germany	chestnut				GG	
R-117	Blind test, KASP	North-West Germany	chestnut				GG	
R-118	Blind test, KASP	North-West Germany	chestnut				GG	

R-119	Blind test, KASP	North-West Germany	chestnut				GG	
R-152	Blind test, KASP	North-West Germany	chestnut				GG	
R-153	Blind test, KASP	North-West Germany	chestnut				GG	
R-154	Blind test, KASP	North-West Germany	chestnut				GG	
R-155	Blind test, KASP	North-West Germany	chestnut				GG	
R-187	Blind test, KASP	North-West Germany	chestnut				GG	
R-190	Blind test, KASP	North-West Germany	chestnut				GG	
R-192	Blind test, KASP	North-West Germany	chestnut				GG	
R-193	Blind test, KASP	North-West Germany	chestnut				GG	
R-224	Blind test, KASP	North-West Germany	chestnut				GT	
R-225	Blind test, KASP	North-West Germany	chestnut				GG	
R-227	Blind test, KASP	North-West Germany	chestnut				GG	
R-249	Blind test, KASP	North-West Germany	chestnut				GG	
R-250	Blind test, KASP	North-West Germany	chestnut				GG	
R-251	Blind test, KASP	North-West Germany	chestnut				GG	
V0055	Blind test, KASP	North-West Germany	chestnut				GT	
V0056	Blind test, KASP	North-West Germany	chestnut				GT	
V0057	Blind test, KASP	North-West Germany	chestnut				GT	
V0059	Blind test, KASP	North-West Germany	chestnut				GT	
V0088	Blind test, KASP	North-West Germany	chestnut				GG	
V0090	Blind test, KASP	North-West Germany	chestnut				GT	
V0092	Blind test, KASP	North-West Germany	chestnut				GG	
V0094	Blind test, KASP	North-West Germany	chestnut				GG	
V0096	Blind test, KASP	North-West Germany	chestnut				GG	
V0097	Blind test, KASP	North-West Germany	chestnut				GT	
V0098	Blind test, KASP	North-West Germany	chestnut				GG	
V0099	Blind test, KASP	North-West Germany	chestnut				GT	
V0100	Blind test, KASP	North-West Germany	chestnut				GG	
V0102	Blind test, KASP	North-West Germany	chestnut				GG	
V0104	Blind test, KASP	North-West Germany	chestnut				GG	
V0105	Blind test, KASP	North-West Germany	chestnut				GG	
V0108	Blind test, KASP	North-West Germany	chestnut				GG	
V0109	Blind test, KASP	North-West Germany	chestnut				GG	
V0111	Blind test, KASP	North-West Germany	chestnut				GG	
V0113	Blind test, KASP	North-West Germany	chestnut				GG	
V0116	Blind test, KASP	North-West Germany	chestnut				GG	
V0117	Blind test, KASP	North-West Germany	chestnut				GG	
V0118	Blind test, KASP	North-West Germany	chestnut				GG	
V0119	Blind test, KASP	North-West Germany	chestnut				GG	
V0120	Blind test, KASP	North-West Germany	chestnut				GG	
V0121	Blind test, KASP	North-West Germany	chestnut				GG	
V0122	Blind test, KASP	North-West Germany	chestnut				GG	
V0124	Blind test, KASP	North-West Germany	chestnut				GG	

V0125	Blind test, KASP	North-West Germany	chestnut				GG	
V0126	Blind test, KASP	North-West Germany	chestnut				GG	
V0128	Blind test, KASP	North-West Germany	chestnut				GG	
V0129	Blind test, KASP	North-West Germany	chestnut				GG	
V0130	Blind test, KASP	North-West Germany	chestnut				GG	
V0132	Blind test, KASP	North-West Germany	chestnut				GG	
V0133	Blind test, KASP	North-West Germany	chestnut				GG	
V0134	Blind test, KASP	North-West Germany	chestnut				GG	
V0137	Blind test, KASP	North-West Germany	chestnut				GT	
V0139	Blind test, KASP	North-West Germany	chestnut				GG	
V0142	Blind test, KASP	North-West Germany	chestnut				GG	
V0143	Blind test, KASP	North-West Germany	chestnut				GG	
V0144	Blind test, KASP	North-West Germany	chestnut				GG	
V0145	Blind test, KASP	North-West Germany	chestnut				GG	
V0146	Blind test, KASP	North-West Germany	chestnut				GG	
V0148	Blind test, KASP	North-West Germany	chestnut				GG	
V0149	Blind test, KASP	North-West Germany	chestnut				GG	
V0150	Blind test, KASP	North-West Germany	chestnut				GG	
V0151	Blind test, KASP	North-West Germany	chestnut				GG	
V0153	Blind test, KASP	North-West Germany	chestnut				GG	
V0154	Blind test, KASP	North-West Germany	chestnut				GG	
V0155	Blind test, KASP	North-West Germany	chestnut				GG	
V0156	Blind test, KASP	North-West Germany	chestnut				GG	
V0157	Blind test, KASP	North-West Germany	chestnut				GG	
V0158	Blind test, KASP	North-West Germany	chestnut				GG	
V0159	Blind test, KASP	North-West Germany	chestnut				GG	
V0160	Blind test, KASP	North-West Germany	chestnut				GG	
V0161	Blind test, KASP	North-West Germany	chestnut				GG	
V0162	Blind test, KASP	North-West Germany	chestnut				GG	
V0163	Blind test, KASP	North-West Germany	chestnut				GG	
V0164	Blind test, KASP	North-West Germany	chestnut				GG	
V0166	Blind test, KASP	North-West Germany	chestnut				GG	
V0168	Blind test, KASP	North-West Germany	chestnut				GT	
V0170	Blind test, KASP	North-West Germany	chestnut				GG	
V0171	Blind test, KASP	North-West Germany	chestnut				GG	
V0172	Blind test, KASP	North-West Germany	chestnut				GG	
V0173	Blind test, KASP	North-West Germany	chestnut				GT	
V0176	Blind test, KASP	North-West Germany	chestnut				GG	
V0177	Blind test, KASP	North-West Germany	chestnut				GT	
V0185	Blind test, KASP	North-West Germany	chestnut				GG	
V0187	Blind test, KASP	North-West Germany	chestnut				GG	
V0189	Blind test, KASP	North-West Germany	chestnut				GG	
V0191	Blind test, KASP	North-West Germany	chestnut				GT	

V0192	Blind test, KASP	North-West Germany	chestnut				GG	
V0194	Blind test, KASP	North-West Germany	chestnut				GG	
V0195	Blind test, KASP	North-West Germany	chestnut				GG	
V0197	Blind test, KASP	North-West Germany	chestnut				GG	
V0200	Blind test, KASP	North-West Germany	chestnut				GG	
V0201	Blind test, KASP	North-West Germany	chestnut				GT	
V0203	Blind test, KASP	North-West Germany	chestnut				GG	
V0206	Blind test, KASP	North-West Germany	chestnut				GG	
V0209	Blind test, KASP	North-West Germany	chestnut				GG	
V0210	Blind test, KASP	North-West Germany	chestnut				GG	
V0211	Blind test, KASP	North-West Germany	chestnut				GG	
V0212	Blind test, KASP	North-West Germany	chestnut				GT	
V0213	Blind test, KASP	North-West Germany	chestnut				GT	
V0214	Blind test, KASP	North-West Germany	chestnut				GG	
V0215	Blind test, KASP	North-West Germany	chestnut				GG	
V0216	Blind test, KASP	North-West Germany	chestnut				GG	
V0217	Blind test, KASP	North-West Germany	chestnut				GG	
V0218	Blind test, KASP	North-West Germany	chestnut				GG	
V0219	Blind test, KASP	North-West Germany	chestnut				GG	
V0220	Blind test, KASP	North-West Germany	chestnut				GG	
V0221	Blind test, KASP	North-West Germany	chestnut				GG	
V0222	Blind test, KASP	North-West Germany	chestnut				GG	
V0223	Blind test, KASP	North-West Germany	chestnut				GG	
V0224	Blind test, KASP	North-West Germany	chestnut				GG	
V0226	Blind test, KASP	North-West Germany	chestnut				GG	
V0228	Blind test, KASP	North-West Germany	chestnut				GG	
V0231	Blind test, KASP	North-West Germany	chestnut				GG	
V0232	Blind test, KASP	North-West Germany	chestnut				GG	
V0233	Blind test, KASP	North-West Germany	chestnut				GG	
V0234	Blind test, KASP	North-West Germany	chestnut				GG	
V0236	Blind test, KASP	North-West Germany	chestnut				GG	
V0239	Blind test, KASP	North-West Germany	chestnut				GG	
V0240	Blind test, KASP	North-West Germany	chestnut				GG	
V0242	Blind test, KASP	North-West Germany	chestnut				GG	
V0244	Blind test, KASP	North-West Germany	chestnut				GG	
V0245	Blind test, KASP	North-West Germany	chestnut				GG	
V0246	Blind test, KASP	North-West Germany	chestnut				GG	
V0248	Blind test, KASP	North-West Germany	chestnut				GG	
V0249	Blind test, KASP	North-West Germany	chestnut				GT	
V0251	Blind test, KASP	North-West Germany	chestnut				GG	
V0252	Blind test, KASP	North-West Germany	chestnut				GG	
V0253	Blind test, KASP	North-West Germany	chestnut				GG	
V0255	Blind test, KASP	North-West Germany	chestnut				GG	

V0256	Blind test, KASP	North-West Germany	chestnut				GG	
V0257	Blind test, KASP	North-West Germany	chestnut				GG	
V0259	Blind test, KASP	North-West Germany	chestnut				GG	
V0261	Blind test, KASP	North-West Germany	chestnut				GG	
V0262	Blind test, KASP	North-West Germany	chestnut				GG	
V0264	Blind test, KASP	North-West Germany	chestnut				GG	
V0268	Blind test, KASP	North-West Germany	chestnut				GG	
V0269	Blind test, KASP	North-West Germany	chestnut				GG	
V0272	Blind test, KASP	North-West Germany	chestnut				GG	
V0273	Blind test, KASP	North-West Germany	chestnut				GG	
V0274	Blind test, KASP	North-West Germany	chestnut				GG	
V0275	Blind test, KASP	North-West Germany	chestnut				GG	
V0276	Blind test, KASP	North-West Germany	chestnut				GG	
V0278	Blind test, KASP	North-West Germany	chestnut				GG	
V0280	Blind test, KASP	North-West Germany	chestnut				GG	
V0282	Blind test, KASP	North-West Germany	chestnut				GG	
V0284	Blind test, KASP	North-West Germany	chestnut				GG	
V0286	Blind test, KASP	North-West Germany	chestnut				GG	
V0289	Blind test, KASP	North-West Germany	chestnut				GG	
V0290	Blind test, KASP	North-West Germany	chestnut				GG	
V0291	Blind test, KASP	North-West Germany	chestnut				GG	
V0292	Blind test, KASP	North-West Germany	chestnut				GG	
V0293	Blind test, KASP	North-West Germany	chestnut				GG	
V0294	Blind test, KASP	North-West Germany	chestnut				GG	
V0295	Blind test, KASP	North-West Germany	chestnut				GG	
V0296	Blind test, KASP	North-West Germany	chestnut				GG	
V0297	Blind test, KASP	North-West Germany	chestnut				GG	
V0299	Blind test, KASP	North-West Germany	chestnut				GG	
V0300	Blind test, KASP	North-West Germany	chestnut				GG	
V0301	Blind test, KASP	North-West Germany	chestnut				GG	
V0302	Blind test, KASP	North-West Germany	chestnut				GG	
V0304	Blind test, KASP	North-West Germany	chestnut				GG	
V0305	Blind test, KASP	North-West Germany	chestnut				GG	
V0306	Blind test, KASP	North-West Germany	chestnut				GG	
V0308	Blind test, KASP	North-West Germany	chestnut				GG	
V0309	Blind test, KASP	North-West Germany	chestnut				GG	
V0312	Blind test, KASP	North-West Germany	chestnut				GG	
V0314	Blind test, KASP	North-West Germany	chestnut				GG	
V0316	Blind test, KASP	North-West Germany	chestnut				GG	
V0317	Blind test, KASP	North-West Germany	chestnut				GG	
V0319	Blind test, KASP	North-West Germany	chestnut				GG	
V0321	Blind test, KASP	North-West Germany	chestnut				GG	
V0322	Blind test, KASP	North-West Germany	chestnut				GG	

V0323	Blind test, KASP	North-West Germany	chestnut				GG		
V0324	Blind test, KASP	North-West Germany	chestnut				GG		
V0327	Blind test, KASP	North-West Germany	chestnut				GG		
V0328	Blind test, KASP	North-West Germany	chestnut				GG		
V0330	Blind test, KASP	North-West Germany	chestnut				GG		
V0331	Blind test, KASP	North-West Germany	chestnut				GG		
V0333	Blind test, KASP	North-West Germany	chestnut				GG		
V0334	Blind test, KASP	North-West Germany	chestnut				GG		
V0335	Blind test, KASP	North-West Germany	chestnut				GG		
V0337	Blind test, KASP	North-West Germany	chestnut				GG		
V0338	Blind test, KASP	North-West Germany	black				TT		
V0339	Blind test, KASP	North-West Germany	chestnut				GG		
V0340	Blind test, KASP	North-West Germany	chestnut				GG		
V0341	Blind test, KASP	North-West Germany	chestnut				GG		
V0343	Blind test, KASP	North-West Germany	chestnut				GG		
V0344	Blind test, KASP	North-West Germany	chestnut				GG		
V0345	Blind test, KASP	North-West Germany	chestnut				GG		
V0346	Blind test, KASP	North-West Germany	chestnut				GG		
V0347	Blind test, KASP	North-West Germany	chestnut				GT		
V0349	Blind test, KASP	North-West Germany	chestnut				GG		
V0351	Blind test, KASP	North-West Germany	chestnut				GG		
V0352	Blind test, KASP	North-West Germany	chestnut				GG		
V0353	Blind test, KASP	North-West Germany	chestnut				GT		
V0354	Blind test, KASP	North-West Germany	chestnut				GG		
V0355	Blind test, KASP	North-West Germany	chestnut				GG		
V0357	Blind test, KASP	North-West Germany	chestnut				GG		
V0358	Blind test, KASP	North-West Germany	chestnut				GG		
V0360	Blind test, KASP	North-West Germany	chestnut				GG		
V0361	Blind test, KASP	North-West Germany	chestnut				GG		
V0365	Blind test, KASP	North-West Germany	chestnut				GT		
V0366	Blind test, KASP	North-West Germany	chestnut				GG		
V0367	Blind test, KASP	North-West Germany	chestnut				GG		
V0368	Blind test, KASP	North-West Germany	chestnut				GG		
V0369	Blind test, KASP	North-West Germany	chestnut				GG		
V0370	Blind test, KASP	North-West Germany	chestnut				GG		
V0371	Blind test, KASP	North-West Germany	chestnut				GG		
V0372	Blind test, KASP	North-West Germany	chestnut				GG		
V0373	Blind test, KASP	North-West Germany	chestnut				GG		
V0374	Blind test, KASP	North-West Germany	chestnut				GG		
V0375	Blind test, KASP	North-West Germany	chestnut				GG		
V0376	Blind test, KASP	North-West Germany	chestnut				GG		
V0377	Blind test, KASP	North-West Germany	chestnut				GG		
V0378	Blind test, KASP	North-West Germany	chestnut				GG		

V0379	Blind test, KASP	North-West Germany	chestnut				GG	
V0380	Blind test, KASP	North-West Germany	chestnut				GG	
V0381	Blind test, KASP	North-West Germany	chestnut				GG	
V0384	Blind test, KASP	North-West Germany	chestnut				GG	
V0386	Blind test, KASP	North-West Germany	chestnut				GG	
V0387	Blind test, KASP	North-West Germany	chestnut				GT	
V0388	Blind test, KASP	North-West Germany	chestnut				GG	
V0389	Blind test, KASP	North-West Germany	chestnut				GG	
V0390	Blind test, KASP	North-West Germany	chestnut				GT	
V0391	Blind test, KASP	North-West Germany	chestnut				GG	
V0393	Blind test, KASP	North-West Germany	chestnut				GG	
V0395	Blind test, KASP	North-West Germany	chestnut				GG	
V0396	Blind test, KASP	North-West Germany	chestnut				GG	
V0397	Blind test, KASP	North-West Germany	chestnut				GG	
V0398	Blind test, KASP	North-West Germany	chestnut				GG	
V0399	Blind test, KASP	North-West Germany	chestnut				GG	
V0400	Blind test, KASP	North-West Germany	chestnut				GG	
V0401	Blind test, KASP	North-West Germany	chestnut				GG	
V0402	Blind test, KASP	North-West Germany	chestnut				GG	
V0403	Blind test, KASP	North-West Germany	chestnut				GG	
V0404	Blind test, KASP	North-West Germany	chestnut				GG	
V0405	Blind test, KASP	North-West Germany	chestnut				GG	
V0406	Blind test, KASP	North-West Germany	chestnut				GG	
V0407	Blind test, KASP	North-West Germany	chestnut				GG	
V0408	Blind test, KASP	North-West Germany	chestnut				GG	
V0410	Blind test, KASP	North-West Germany	chestnut				GG	
V0411	Blind test, KASP	North-West Germany	chestnut				GG	
V0412	Blind test, KASP	North-West Germany	chestnut				GG	
V0413	Blind test, KASP	North-West Germany	chestnut				GG	
V0414	Blind test, KASP	North-West Germany	chestnut				GG	
V0415	Blind test, KASP	North-West Germany	chestnut				GG	
V0416	Blind test, KASP	North-West Germany	chestnut				GG	
V0417	Blind test, KASP	North-West Germany	chestnut				GG	
V0420	Blind test, KASP	North-West Germany	chestnut				GG	
V0423	Blind test, KASP	North-West Germany	chestnut				GG	
V0424	Blind test, KASP	North-West Germany	chestnut				GG	
V0425	Blind test, KASP	North-West Germany	chestnut				GG	
V0427	Blind test, KASP	North-West Germany	chestnut				GG	
V0429	Blind test, KASP	North-West Germany	chestnut				GG	
V0432	Blind test, KASP	North-West Germany	chestnut				GG	
V0433	Blind test, KASP	North-West Germany	chestnut				GG	
V0594	Blind test, KASP	North-West Germany	black				TT	
V0595	Blind test, KASP	North-West Germany	chestnut				GG	

V0036	Blind test, KASP	Saxony-Anhalt	black					TT		
V0037	Blind test, KASP	Saxony-Anhalt	black					TT		
V0063	Blind test, KASP	Saxony-Anhalt	chestnut					GG		
V0064	Blind test, KASP	Saxony-Anhalt	chestnut					GG		
V0065	Blind test, KASP	Saxony-Anhalt	chestnut					GG		
V0066	Blind test, KASP	Saxony-Anhalt	chestnut					GG		
V0067	Blind test, KASP	Saxony-Anhalt	chestnut					GG		
V0068	Blind test, KASP	Saxony-Anhalt	chestnut					GG		
V0069	Blind test, KASP	Saxony-Anhalt	chestnut					GG		
V0070	Blind test, KASP	Saxony-Anhalt	chestnut					GG		
V0071	Blind test, KASP	Saxony-Anhalt	chestnut					GG		
V0072	Blind test, KASP	Saxony-Anhalt	chestnut					GG		
V0073	Blind test, KASP	Saxony-Anhalt	chestnut					GG		
V0074	Blind test, KASP	Saxony-Anhalt	chestnut					GG		
V0075	Blind test, KASP	Saxony-Anhalt	chestnut					GG		
V0076	Blind test, KASP	Saxony-Anhalt	chestnut					GG		
V0077	Blind test, KASP	Saxony-Anhalt	chestnut					GG		
V0078	Blind test, KASP	Saxony-Anhalt	chestnut					GG		
V0079	Blind test, KASP	Saxony-Anhalt	chestnut					GG		
V0080	Blind test, KASP	Saxony-Anhalt	chestnut					GG		
V0081	Blind test, KASP	Saxony-Anhalt	chestnut					GG		
V0082	Blind test, KASP	Saxony-Anhalt	chestnut					GG		
V0083	Blind test, KASP	Saxony-Anhalt	chestnut					GG		
V0084	Blind test, KASP	Saxony-Anhalt	chestnut					GG		
V0085	Blind test, KASP	Saxony-Anhalt	chestnut					GG		
V0086	Blind test, KASP	Saxony-Anhalt	chestnut					GG		
V0087	Blind test, KASP	Saxony-Anhalt	chestnut					GG		

© 2020 by the authors. Submitted for possible open access publication under the terms and conditions of the Creative Commons Attribution (CC BY) license (http://creativecommons.org/licenses/by/4.0/).
